# SPI1 Promotes TNF‐α–Induced Epithelial–Mesenchymal Transition–Like Changes in Retinal Pigment Epithelial Cells: Implications for the Pathogenesis of Age‐Related Macular Degeneration

**DOI:** 10.1155/joph/8106992

**Published:** 2026-07-29

**Authors:** Xingyi Zhang, Hua Liu

**Affiliations:** ^1^ Department of Ophthalmology, Dalian Medical University, Dalian 116000, China, dlmedu.edu.cn

**Keywords:** age-related macular degeneration, ALKBH5, epithelial–mesenchymal transition, retinal pigment epithelial cells, SPI1

## Abstract

**Background/Aims:**

Age‐related macular degeneration (AMD) is a progressive degenerative disease of the retinal macula associated with aging, which is one of the main causes of vision loss in the elderly. This research aims to investigate the mechanism of SPI1 in EMT of RPE cells in AMD, providing new targets for AMD treatment.

**Methods:**

SPI1, ALKBH5, and NURR1 expression was assayed by RT‐qPCR and Western blot in AMD patients and cell models. The relationship between SPI1 expression and clinical features of AMD patients was analyzed, and the correlations among SPI1, ALKBH5, and NURR1 were analyzed. The diagnostic value of SPI1 in AMD was verified via ROC curve. ROS levels were detected. N‐cadherin and E‐cadherin were detected by Western blot. Cell migration was detected by transwell assay. The binding of SPI1 to ALKBH5 in cells was verified. m6A levels on NURR1 were detected.

**Results:**

SPI1 and ALKBH5 were highly expressed, while NURR1 was lowly expressed in TNF‐α–induced human RPE cells. SPI1 expression was correlated with age and staging of AMD patients. SPI1 expression exhibited a positive association with ALKBH5 expression, while showing a negative association with NURR1 expression. After downregulation of SPI1, N‐cadherin was downregulated, E‐cadherin was upregulated, ROS levels were decreased, and cell migration was reduced. SPI1 promoted ALKBH5 expression, and ALKBH5 downregulated NURR1 expression via m6A modification. ALKBH5 overexpression or NURR1 downregulation partially reversed the attenuating effect of SPI1 downregulation on EMT‐like changes in RPE cells.

**Conclusion:**

SPI1 stimulates EMT‐like changes of RPE cells via the ALKBH5/NURR1 axis. This pathway may participate in AMD‐related pathological processes.

## 1. Introduction

Age‐related macular degeneration (AMD) ranks as a leading cause of blindness in elderly individuals and is characterized by pathological changes in the deep retinal layers of the macular region and the surrounding blood vessels leading to central vision loss [1]. During aging, retinal pigment epithelial (RPE) cells become insufficient to decompose and clear metabolic waste that accumulates as a result of the extremely high metabolic activity in the macular region, thereby contributing to the development of AMD [2]. Under the pathological microenvironment of AMD, RPE cells initiate epithelial–mesenchymal transition (EMT), differentiate into myofibroblasts, and participate in the formation of subretinal fibrosis, thereby exacerbating AMD progression [3]. Novel therapeutic approaches that target AMD from the perspective of EMT, particularly with respect to retinal structural disorder, have emerged [4]; however, the underlying mechanisms remain to be further elucidated.

Spi‐1 proto‐oncogene (SPI1), a transcription factor, is primarily expressed in hematopoietic cells and lymphocytes and is recognized as a key regulator of myeloid cell development and the terminal differentiation of macrophages [5]. A recent study has demonstrated that SPI1 expression is upregulated in mouse models of AMD and contributes to inflammatory responses and pathological neovascularization [6]. Silencing SPI1 attenuates oxidative stress and mitochondrial dysfunction in RPE cells and rescues retinal structure [7]. Although SPI1 has been identified as a transcription factor that regulates EMT‐related genes [8], the mechanism by which it contributes to AMD via EMT remains unclear.

N6‐methyladenosine (m6A) is the most widespread, abundant, and conserved internal co‐transcriptional modification in eukaryotic RNA and influences disease progression [9]. AlkB homolog 5 (ALKBH5) is one of the demethylases involved in RNA m6A modification, which can remove m6A modifications, and is associated with processes such as metabolism, immunity, and reproduction [10]. Accumulating evidence indicates that m6A modification serves as a critical player in the EMT process of RPE cells in AMD, and dysregulated m6A modification contributes to impairments in RPE function and structure [11, 12]. The JASPAR database (https://jaspar.elixir.no/) [13] predicts that SPI1 can bind to the ALKBH5 promoter. We hypothesize that ALKBH5 regulates the EMT progression of RPE cells in AMD through demethylation, thereby participating in the pathogenesis of AMD.

Nuclear receptor subfamily 4 group A member 2 (NURR1) is a nuclear receptor transcription factor responsible for the development, regulation, and maintenance of dopaminergic neurons in the brain [14]. A previous study demonstrated that NURR1 protein expression is a key factor in retinal development [15]. Interestingly, the 3′‐untranslated region (UTR) of NURR1 mRNA contains abundant m6A binding sites [16]. Therefore, we speculate that ALKBH5 downregulates the expression of NURR1 through m6A modification.

This research seeks to elucidate the association between SPI1 and the clinicopathological characteristics of AMD patients and to investigate the underlying mechanisms using an AMD cell model established by inducing EMT in human RPE cells with tumor necrosis factor (TNF)‐α, thereby providing a novel theoretical basis for AMD treatment.

## 2. Materials and Methods

### 2.1. Ethics Statement

This research was approved by the Academic Ethics Committee of Dalian Medical University. All patients were fully informed of the purpose of the study and signed the informed consent form before sampling.

### 2.2. Study Subjects

This study prospectively enrolled 68 AMD cases who visited Dalian Medical University from January 2020 to December 2023 as research subjects and 68 healthy volunteers as control.

### 2.3. Inclusion and Exclusion Criteria

The inclusion criteria for the AMD group included the following [17]: (1) clinical diagnosis of AMD; (2) age ≥ 50 years; (3) clear media; and (4) good pupil dilation.

The inclusion criteria for the healthy control group included the following: (1) age ≥ 50 years and (2) no ocular diseases other than refractive error or a prior history of cataract surgery.

The AMD staging criteria included the following [18]: (1) early AMD: medium drusen > 63 μm and ≤ 125 μm, without pigment abnormalities; (2) intermediate AMD: large drusen > 125 μm and/or pigment abnormalities; and (3) late AMD: neovascular AMD and/or any geographic atrophy.

The exclusion criteria for AMD included the following [17]: (1) patients who have ocular diseases other than AMD that affect visual function or ocular morphology and (2) patients who are unable to undergo all ophthalmic examinations.

### 2.4. Clinical Data Collection

Data of the study population at enrollment were recorded, including gender, age, smoking status (smoking/nonsmoking), and staging (early/intermediate/late).

### 2.5. Serum Sample Collection

Peripheral venous blood (5 mL) was collected from all subjects in the early morning of the second day after admission under fasting conditions. The blood was centrifuged at 1500 × *g*, 4°C for 10 min using a centrifuge. The supernatant serum was harvested and stored in a −80°C cryogenic refrigerator for later use.

### 2.6. Cell Culture and Treatment

The human RPE cell line ARPE19 was procured from ATCC (Manassas, VA, USA). All cells were cultured in Dulbecco’s Modified Eagle Medium (DMEM; Invitrogen, Carlsbad, CA, USA) containing 10% fetal bovine serum (FBS, Gibco, Grand Island, NY, USA), 100 U/mL penicillin, and 100 μg/mL streptomycin at 37°C under 5% CO_2_. Based on a previous study [19], ARPE19 cells were treated with TNF‐α (10 ng/mL) for 24 h to establish the AMD cell model.

After 24 h of cell culture, the cells were seeded in 6‐well plates. ARPE19 cells were transiently transfected using Lipofectamine 2000 (Invitrogen) with the following: (i) si‐SPI1 (si‐SPI1#1, si‐SPI1#2) or its negative control si‐NC; (ii) oe‐ALKBH5 or oe‐SPI1 (both packaged in the pcDNA3.1 vector) or their negative control oe‐NC; and (iii) si‐NURR1 (si‐NURR1#1, si‐NURR1#2) or its negative control si‐NC. All plasmids and the corresponding empty vectors were provided by GenePharma (Shanghai, China). Subsequent experiments were performed 48 h after cell transfection.

### 2.7. Reverse‐Transcription Quantitative Polymerase Chain Reaction (RT‐qPCR)

The expression of SPI1, ALKBH5, and NURR1 in cells and serum was detected by RT‐qPCR. Briefly, total RNA extraction from ARPE19 cells or serum was conducted using the TRIzol kit (Invitrogen). cDNA was obtained by reverse transcription using the PrimeScript RT‐PCR kit (Takara, Tokyo, Japan). qPCR was performed using TB Green Premix Ex Taq II (Takara, RR820A), and gene expression was measured using a real‐time fluorescent quantitative PCR system (Applied Biosystems 7500, Carlsbad, California, USA). With glyceraldehyde‐3‐phosphate dehydrogenase (GAPDH) as the endogenous control for cellular samples and 18S rRNA as the reference gene for serum samples, the relative gene expression was calculated by the 2^−ΔΔCt^ method. The primer sequences are listed in Table 1.

**TABLE 1:** qPCR primers.

Gene	Sequences (5′‐3′)
SPI1	F: AATCAGGAACTTGTGCTGGC
	R: AACAGGAACTGGTACAGGCG

ALKBH5	F: AGTTCAGTCTTCTGCTCGCC
	R: AGGAACTGTGGACATGGCAG

NURR1	F: TGGACTCCCCATTGCTTTTCT
	R: GGACAGGGGCATTTGGTACA

18S rRNA	F: TGCCCTATCAACTTTCGATGGTAGTC
	R: TTGGATGTGGTAGCCGTTTCTCA

GAPDH	F: GATGCTGGCGCTGAGTACG
	R: GCTAAGCAGTTGGTGGTGC

### 2.8. Western Blot

Total protein extraction from cells or serum was conducted using radioimmunoprecipitation assay (RIPA) lysis buffer (Thermo Fisher Scientific, Waltham, MA, USA). The extracted proteins were quantified via a bicinchoninic acid (BCA) protein assay kit (Thermo Fisher Scientific). Proteins were separated on 12% sodium dodecyl sulfate polyacrylamide gel electrophoresis (SDS‐PAGE) and transferred onto polyvinylidene fluoride (PVDF) membranes. Blocking of the membranes was conducted with 5% skimmed milk at room temperature for 1 h. Following washing with phosphate‐buffered saline (PBS), the membranes were incubated overnight at 4°C with the following primary antibodies: anti‐SPI1 (ab302623, 1:1000, Abcam, Cambridge, UK), anti‐ALKBH5 (ab195377, 1:1000, Abcam), anti‐NURR1 (NURR1‐101AP, 1:1000, Thermo Fisher Scientific), anti‐N‐cadherin (ab76011, 1:5000, Abcam), anti‐E‐cadherin (ab40772, 1:1000, Abcam), anti‐CD81 (ab155760, 1:1000, Abcam), and anti‐GAPDH (ab9485, 1:2500, Abcam). Next day, the membranes were washed thrice with Tris‐buffered saline with Tween 20 (TBST) and then incubated with secondary antibody immunoglobulin G (IgG) (ab6721, 1:2000, Abcam) at room temperature for 1 h. After rinsing with PBS, signal visualization was performed using the enhanced chemiluminescence (ECL) method (Sigma‐Aldrich, St. Louis, MO, USA), and relevant quantification was conducted using ImageJ software. GAPDH was adopted as the loading control for cellular proteins, and CD81 served as the loading control for serum proteins. Each serum sample was individually analyzed by Western blot.

### 2.9. Detection of Reactive Oxygen Species (ROS) Levels

The ROS level in cells was assayed via a ROS kit (E004‐1‐1, Nanjing Jiancheng Bioengineering Institute, Nanjing, Jiangsu, China). Briefly, 2′,7′‐dichlorofluorescin diacetate (DCFH‐DA) was supplemented to the culture medium for a 30‐min incubation at 37°C. After centrifugation, cell pellets were collected and then resuspended in PBS. Fluorescence intensity changes of each well were measured at 525 nm using a Bio‐Rad fluorescence microplate reader. All results were presented as relative fluorescence intensity normalized to the control group.

### 2.10. Transwell Assay

ARPE19 cells (1 × 10^6^ cells/well) were inoculated into 6‐well plates. After treatment with TNF‐α for 24 h, ARPE19 cells were subjected to trypsinization and resuspension. ARPE19 cells (1 × 10^4^ cells/well) were plated into the upper insert of transwell chambers (8‐μm pores; Falcon; Corning Life Sciences, Corning, NY, USA). Complete medium (500 μL) was supplemented to the lower well. Following incubation at 37°C for 48 h, cells in the upper insert were wiped off with a swab. The migrated cells (penetrating the pores) were fixed in cold methanol for 10 min and stained using 0.5% crystal violet solution (Beyotime, Shanghai, China) for 1 h. Migrated RPE cells were imaged via a Leica microscope (DMI3000, Germany), followed by quantification via ImageJ software. Migrated cells were calculated.

### 2.11. Bioinformatics

The binding of SPI1 to the ALKBH5 promoter was predicted using the JASPAR database (https://jaspar.elixir.no/) [13].

### 2.12. Dual‐Luciferase Assay

Synthesized ALKBH5 promoter sequences containing the SPI1 binding site (ALKBH5‐WT) and those with mutations at the binding site (ALKBH5‐MUT) were, respectively, cloned into dual‐luciferase reporter plasmids (GenePharma). The constructed luciferase reporter plasmids were co‐transfected into ARPE19 cells with oe‐NC or oe‐SPI1, respectively. After 48 h of incubation, luciferase activity was assayed via the dual‐luciferase reporter assay system (Promega, Madison, WI, USA).

### 2.13. Chromatin Immunoprecipitation (ChIP)

To verify the binding of SPI1 to the ALKBH5 promoter, ChIP was conducted via the EZ‐ChIP kit (Millipore, Billerica, MA, USA). Briefly, treated or untreated ARPE19 cells were crosslinked with formaldehyde, and DNA fragments were obtained by sonication. Immunoprecipitation was then conducted using anti‐SPI1 antibody (ab302623, Abcam) or anti‐IgG antibody (ab6757, Abcam). DNA from the immunoprecipitated DNA‐protein complexes was extracted, purified, and detected via RT‐qPCR, with GAPDH as a negative control. The primer sequences for the ALKBH5 promoter region were as follows: forward primer: 5′‐TGCCTGATTGACACGCATGA‐3′; reverse primer: 5′‐TCTACAGGGGTGCGCTCATA‐3′.

### 2.14. m6A Quantitative Analysis

RNA extraction from serum or ARPE19 cells was conducted to determine the total m6A modification levels. The total m6A content in RNA was assayed via the m6A RNA Methylation Quantification Kit (ab185912; Abcam). The total m6A content in RNA was measured by absorbance at 450 nm.

### 2.15. Methylated RNA Immunoprecipitation (MeRIP)‐qPCR

NURR1 m6A level was assessed via MeRIP‐qPCR. Briefly, total RNA was isolated from serum or ARPE19 cells. Chemically fragmented RNA was incubated with m6A antibody (ab286164, Abcam) for immunoprecipitation via the Magna Methylated RNA Immunoprecipitation m6A kit (Millipore). The immunoprecipitated m6A RNA was recovered via elution buffer, and m6A enrichment on NURR1 was analyzed via RT‐qPCR.

### 2.16. RNA Stability Analysis

Cells were treated with actinomycin D, an RNA synthesis inhibitor, at a concentration of 5 μg/mL. Total RNA was extracted using TRIzol reagent at different time points (0, 4, 8, and 12 h) after treatment. The harvested RNA was reverse‐transcribed into cDNA with the iScript cDNA Synthesis Kit (Bio‐Rad, Hercules, CA, USA). Subsequently, the half‐life of NURR1 mRNA was quantified by RT‐qPCR using SYBR Green Supermix (Bio‐Rad), and GAPDH was applied as the reference gene for normalization.

### 2.17. Statistical Analysis

Data were statistically analyzed and graphed via SPSS 21.0 software (IBM SPSS Statistics, Chicago, IL, USA) and GraphPad Prism 8.0 software (GraphPad Software Inc., San Diego, CA, USA). Quantitative data were expressed as mean ± standard deviation (SD). Normality and homogeneity of variance tests were first conducted, and data conforming to normal distribution and homogeneous variance were included for subsequent analyses. Comparisons between two groups were assayed via *t*‐test; comparisons among multiple groups were assayed via one‐way or two‐way ANOVA, followed by Tukey’s multiple comparisons test. Receiver operating characteristic (ROC) curve was used to analyze the diagnostic value of SPI1 for AMD patients. Pearson correlation analysis was used to assess the correlations between the expression of SPI1 and that of ALKBH5 or NURR1 in the serum of AMD patients. All tests were two‐tailed, and *p* value < 0.05 was considered statistically significant.

## 3. Results

### 3.1. SPI1 Is Highly Expressed in AMD and Correlates With Clinicopathological Features of AMD Patients

**FIGURE 1:**
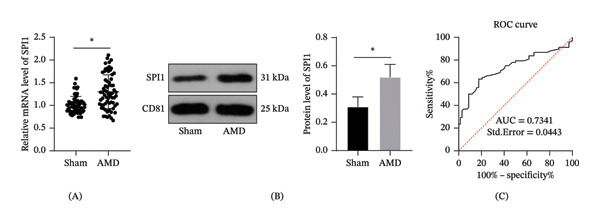
SPI1 is highly expressed in AMD and correlates with clinicopathological characteristics of AMD patients. (A, B) The expression of SPI1 in the serum of clinical subjects was detected by RT‐qPCR and Western blot, and representative blots were shown in (B), with 18S rRNA as the reference gene for RT‐qPCR of serum samples and CD81 as the internal control for Western blot. (C) ROC curve for diagnosing AMD based on serum SPI1 levels; *n* = 68. Data are expressed as mean ± standard deviation. *t* test was used for data analysis in (A) and (B); ^∗^ *p* < 0.05.

A previous study has found that SPI1 is upregulated in AMD [6]. To elucidate the mechanism of SPI1 in AMD, we detected SPI1 expression in the serum of subjects using RT‐qPCR and Western blot. SPI1 expression in AMD patients was higher than the control group (*p* < 0.05, Figure 1A, B). We further constructed an ROC curve to evaluate the diagnostic value of SPI1 expression levels for AMD (Figure 1C), which revealed an area under the curve (AUC) of 0.734 (95% CI: 0.652–0.806) with a cutoff value of 1.14 (sensitivity: 63.24%, specificity: 82.35%), indicating that SPI1 levels show limited clinical value when used as an independent biomarker but can serve as a supplementary indicator. To investigate the relationship between SPI1 expression and clinicopathological features of AMD patients, we stratified 68 AMD patients into SPI1 high‐expression and low‐expression groups using the median expression of SPI1 in the serum of AMD patients as the cutoff value. Collectively, SPI1 expression was associated with age and stage (*p* < 0.05, Table 2).

**TABLE 2:** Correlation between SPI1 expression and clinicopathological characteristics of AMD patients.

Characteristic	Number	SPI1		*p* value
		Low expression (*N* = 34)	High expression (*N* = 34)	
*Gender*				0.330
Male	37	21	16	
Female	31	13	18	

*Age*				0.007
≤ 60	34	23	11	
> 60	34	11	23	

*Stage*				0.015
Early, intermediate	48	29	19	
Late	20	5	15	

*Smoking*				0.330
Yes	37	16	21	
No	31	18	13	

*Note:* Fisher’s exact test was used for comparisons between groups.

### 3.2. Downregulation of SPI1 Attenuates EMT‐Like Changes in TNF‐α–Induced Human RPE Cells

**FIGURE 2:**
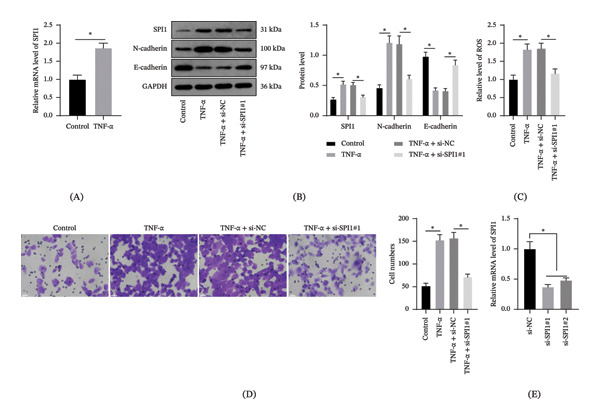
Downregulation of SPI1 attenuates EMT‐like changes in TNF‐α–induced human RPE cells. ARPE19 cells were treated with TNF‐α (10 ng/mL). To downregulate SPI1 levels, ARPE19 cells were transfected with si‐SPI1#1 or si‐SPI1#2, with si‐NC transfection serving as the negative control. (A) RT‐qPCR was used to detect SPI1 expression in ARPE19 cells, with GAPDH as the endogenous internal control. (B) Western blot was performed to determine the expression of SPI1, N‐cadherin, and E‐cadherin in ARPE19 cells, with GAPDH as the endogenous internal control. (C) Kit was used to measure ROS levels in ARPE19 cells. (D) Transwell assay was conducted to assess cell migration. (E) RT‐qPCR was used to detect transfection efficiency. *n* = 3. Data are expressed as mean ± standard deviation. *t* test was used for data analysis in (A); two‐way ANOVA was used for data analysis in (B); one‐way ANOVA was used for data analysis in (C)–(E), with Tukey’s multiple comparisons test as the post hoc test. ^∗^ *p* < 0.05.

To investigate the role of SPI1 in EMT of human RPE cells, we induced EMT‐like changes in human RPE cells (ARPE19) via TNF‐α stimulation. Our analyses showed that TNF‐α treatment significantly upregulated intracellular SPI1 expression (*p* < 0.05, Figure 2A, B), markedly increased N‐cadherin expression while decreasing E‐cadherin expression (*p* < 0.05, Figure 2B), elevated ROS levels (*p* < 0.05, Figure 2C), and enhanced cell migration (*p* < 0.05, Figure 2D). Subsequently, we transfected cells with si‐SPI1#1 or si‐SPI1#2 to downregulate SPI1 levels (*p* < 0.05, Figure 2B, E) and selected si‐SPI1#1 with superior transfection efficiency for subsequent experiments. Following SPI1 downregulation, we observed a significant decrease in N‐cadherin expression alongside a marked increase in E‐cadherin expression (*p* < 0.05, Figure 2B), lowered ROS levels (*p* < 0.05, Figure 2C), and diminished cell migration (*p* < 0.05, Figure 2D). Collectively, downregulation of SPI1 reduces EMT‐like changes in TNF‐α–induced human RPE cells.

### 3.3. SPI1 Transcriptionally Activates ALKBH5 Expression

**FIGURE 3:**
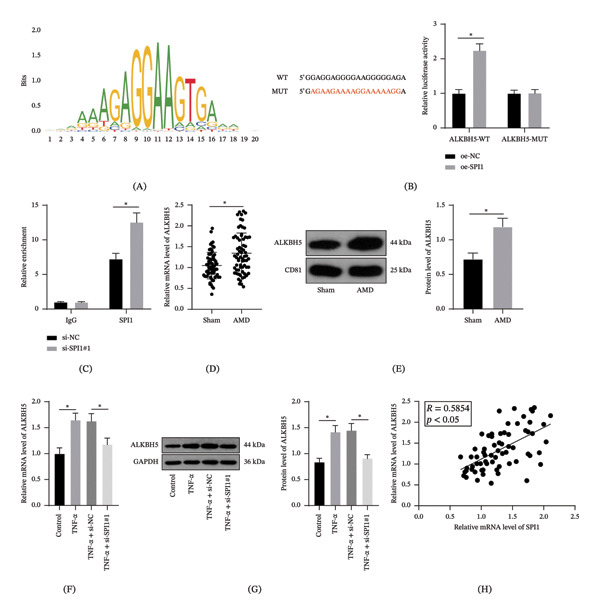
SPI1 transcriptionally activates ALKBH5 expression. (A) Prediction of the binding between SPI1 and the ALKBH5 promoter via the JASPAR database. (B, C) Dual‐luciferase reporter assay and ChIP assay were performed to verify the binding interaction between SPI1 and ALKBH5 (*n* = 3). (D, E) RT‐qPCR and Western blot were used to detect ALKBH5 expression in the serum of clinical subjects, and representative blots were shown in (E) (*n* = 68), with 18S rRNA as the reference gene for RT‐qPCR of serum samples and CD81 as the internal control for Western blot. (F, G) RT‐qPCR and Western blot were conducted to determine ALKBH5 expression in ARPE19 cells (*n* = 3), with GAPDH as the endogenous internal control. (H) Pearson correlation analysis of the correlation between SPI1 and ALKBH5 expression in the serum of 68 AMD patients (*n* = 68). Data are expressed as mean ± standard deviation. Two‐way ANOVA was used for data analysis in (B) and (C); *t* test was used for data analysis in (D) and (E); one‐way ANOVA was used for data analysis in (F) and (G), with Tukey’s multiple comparisons test as the post hoc test. ^∗^ *p* < 0.05.

Studies have demonstrated that SPI1, as a transcription factor, positively regulates the expression of downstream genes by binding to their promoters [20, 21]. The JASPAR database prediction revealed that SPI1 can bind to the ALKBH5 promoter (Figure 3A). ALKBH5 is upregulated in AMD [22], leading us to hypothesize that SPI1 may bind to the ALKBH5 promoter to upregulate its expression. To verify this conjecture, we performed a dual‐luciferase reporter assay, which confirmed a binding interaction between SPI1 and the ALKBH5 promoter (*p* < 0.05, Figure 3B). ChIP further demonstrated that SPI1 was enriched at the ALKBH5 promoter, which was reduced after downregulation of SPI1 (*p* < 0.05, Figure 3C).

Subsequently, we detected ALKBH5 expression in the serum of subjects and ARPE19 cells. The results showed that ALKBH5 was highly expressed in the serum of AMD patients and in TNF‐α–treated ARPE19 cells, while ALKBH5 expression was significantly downregulated following SPI1 silencing (*p* < 0.05, Figure 3D–G). Pearson correlation analysis revealed a positive association between SPI1 and ALKBH5 expression in the serum of AMD patients (Figure 3H). Collectively, SPI1 transcriptionally activates ALKBH5 expression, with a positive correlation between SPI1 and ALKBH5 expression.

### 3.4. ALKBH5 Overexpression Partially Reverses the Attenuating Effect of SPI1 Downregulation on EMT‐Like Changes in Human RPE Cells

**FIGURE 4:**
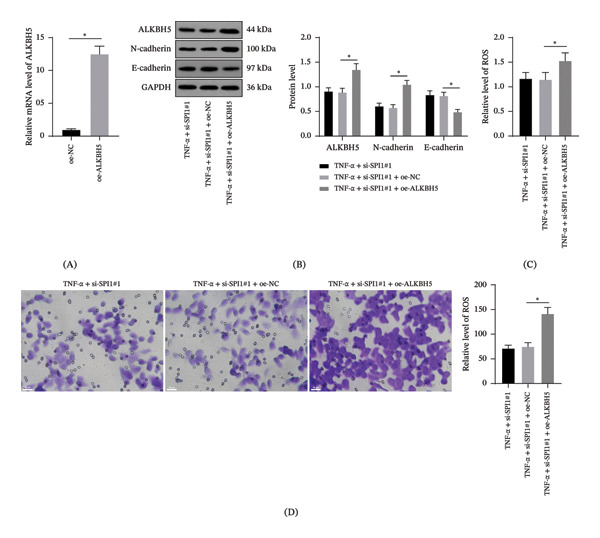
Overexpression of ALKBH5 partially reverses the attenuating effect of SPI1 downregulation on EMT‐like changes in human RPE cells. ARPE19 cells were transfected with oe‐ALKBH5 to upregulate intracellular ALKBH5 levels, with oe‐NC transfection serving as the negative control. Combined experiments were performed with si‐SPI1#1. (A) RT‐qPCR was used to detect ALKBH5 expression in ARPE19 cells, with GAPDH as the endogenous internal control. (B) Western blot was performed to determine the expression of ALKBH5, N‐cadherin, and E‐cadherin in ARPE19 cells, with GAPDH as the endogenous internal control. (C) Kit was used to measure ROS levels in ARPE19 cells. (D) Transwell assay was conducted to assess cell migration. *n* = 3. Data are expressed as mean ± standard deviation. *t* test was used for data analysis in (A); two‐way ANOVA was used for data analysis in (B); one‐way ANOVA was used for data analysis in (C) and (D), with Tukey’s multiple comparisons test as the post hoc test. ^∗^ *p* < 0.05.

To elucidate the role of ALKBH5 in AMD, we transfected ARPE19 cells with oe‐ALKBH5, which successfully upregulated intracellular ALKBH5 levels (*p* < 0.05, Figure 4A, B). A combined experiment with si‐SPI1#1 was then performed. ALKBH5 overexpression significantly upregulated N‐cadherin expression while downregulating E‐cadherin expression (*p* < 0.05, Figure 4B), increased ROS levels (*p* < 0.05, Figure 4C), and enhanced cell migration (*p* < 0.05, Figure 4D). Collectively, ALKBH5 overexpression reverses the attenuating effect of SPI1 downregulation on EMT‐like changes in human RPE cells.

### 3.5. ALKBH5 Eliminates m6A Modifications to Alter NURR1 mRNA Stability and Reduce NURR1 Expression

**FIGURE 5:**
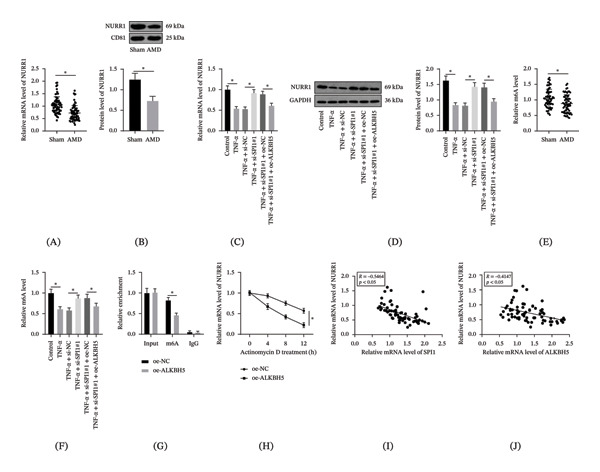
ALKBH5 downregulates NURR1 expression via m6A modification. (A, B) RT‐qPCR and Western blot were used to detect NURR1 expression in the serum of clinical subjects, and representative blots were shown in (B) (*n* = 68), with 18S rRNA as the reference gene for RT‐qPCR of serum samples and CD81 as the internal control for Western blot. (C, D) RT‐qPCR and Western blot were conducted to determine NURR1 expression in ARPE19 cells (*n* = 3), with GAPDH as the endogenous internal control. (E, F) m6A quantitative analysis of m6A levels in serum and ARPE19 cells. (G) MeRIP‐qPCR was performed to detect m6A levels on NURR1 in ARPE19 cells. (H) NURR1 mRNA stability was examined after actinomycin D treatment (*n* = 3), with GAPDH as the endogenous internal control. (I) Pearson correlation analysis of the correlation between SPI1 and NURR1 expression in the serum of 68 AMD patients (*n* = 68). (J) Pearson correlation analysis of the correlation between ALKBH5 and NURR1 expression in the serum of 68 AMD patients (*n* = 68). Data are expressed as mean ± standard deviation. *t* test was used for data analysis in (A), (B), and (E); one‐way ANOVA was used for data analysis in (C), (D), and (F); two‐way ANOVA was used for data analysis in (G) and (H), with Tukey’s multiple comparisons test as the post hoc test. ^∗^ *p* < 0.05.

ALKBH5, as a demethylase, can downregulate downstream genes via m6A modification [23]. Additionally, NURR1 can attenuate TNF‐α–induced EMT in human RPE cells [19]. Thus, we hypothesized that ALKBH5 downregulates NURR1 expression through m6A modification. We detected NURR1 expression in the serum of clinical subjects and ARPE19 cells. NURR1 was lowly expressed in the serum of AMD patients and in TNF‐α–treated ARPE19 cells. NURR1 expression was significantly upregulated after SPI1 downregulation, whereas NURR1 expression was downregulated after ALKBH5 overexpression (*p* < 0.05, Figure 5A–D). Subsequent measurement revealed that m6A levels were decreased in the serum of AMD patients and in TNF‐α–treated ARPE19 cells. m6A levels were significantly increased after SPI1 downregulation but significantly decreased after ALKBH5 overexpression (*p* < 0.05, Figure 5E, F). Moreover, ALKBH5 overexpression led to a significant reduction in m6A enrichment on NURR1 (*p* < 0.05, Figure 5G) and NURR1 mRNA stability (*p* < 0.05, Figure 5H). Pearson correlation analysis demonstrated that SPI1 and ALKBH5 expressions were negatively correlated with NURR1 expression (Figure 5I, J). Collectively, ALKBH5‐mediated m6A modification downregulates NURR1 expression.

### 3.6. Downregulation of NURR1 Partially Reverses the Attenuating Effect of SPI1 Downregulation on EMT‐Like Changes in Human RPE Cells

**FIGURE 6:**
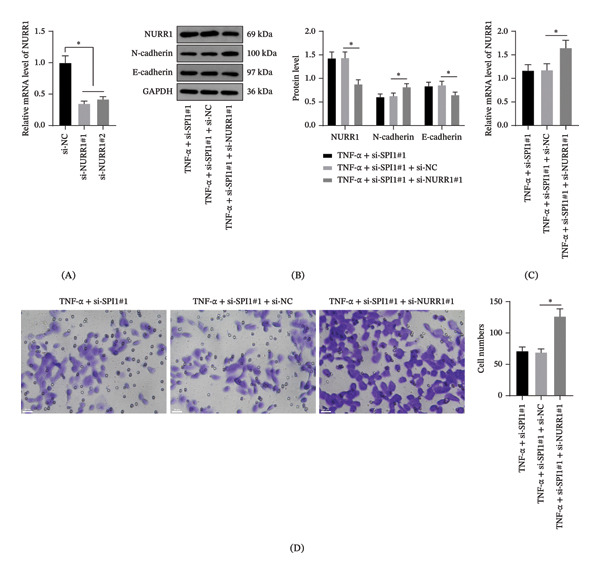
Downregulation of NURR1 partially reverses the attenuating effect of SPI1 downregulation on EMT‐like changes in human RPE cells. ARPE19 cells were transfected with si‐NURR1#1 or si‐NURR1#2 to downregulate intracellular NURR1 levels, with si‐NC transfection serving as the negative control. Combined experiments were performed with si‐SPI1#1. (A) RT‐qPCR was used to detect NURR1 expression in ARPE19 cells, with GAPDH as the endogenous internal control. (B) Western blot was performed to determine the expression of NURR1, N‐cadherin, and E‐cadherin in ARPE19 cells, with GAPDH as the endogenous internal control. (C) Kit was used to measure ROS levels in ARPE19 cells. (D) Transwell assay was conducted to assess cell migration. *n* = 3. Data are expressed as mean ± standard deviation. One‐way ANOVA was used for data analysis in (A), (C), and (D); two‐way ANOVA was used for data analysis in (B), with Tukey’s multiple comparisons test as the post hoc test. ^∗^ *p* < 0.05.

To explore the role of NURR1 in AMD, we transfected ARPE19 cells with si‐NURR1#1 or si‐NURR1#2, which successfully downregulated intracellular NURR1 levels (*p* < 0.05, Figure 6A, B). si‐NURR1#1, with superior transfection efficiency, was employed for combined experiments with si‐SPI1#1. Following NURR1 downregulation, intracellular N‐cadherin expression was upregulated while E‐cadherin expression was downregulated (*p* < 0.05, Figure 6B), ROS levels were increased (*p* < 0.05, Figure 6C), and cell migration was significantly enhanced (*p* < 0.05, Figure 6D). Collectively, downregulation of NURR1 reverses the attenuating effect of SPI1 downregulation on EMT‐like changes in human RPE cells.

## 4. Discussion

AMD is classified into dry (atrophic) and wet (exudative) forms, while atrophic type is characterized by progressive atrophy of the RPE layer and outer retinal layers, and the exudative type is defined by the disordered invasion of the RPE and retina by choroidal neovascularization [1]. Current treatment options for dry and wet AMD, including antioxidants, anti‐inflammatory drugs, anti‐VEGF agents, and laser surgery [24], remain unable to completely cure AMD. This study demonstrated that SPI1 is highly expressed in TNF‐α–induced RPE cells. SPI1 has limited clinical utility as an independent biomarker but may serve as a complementary diagnostic indicator for AMD. SPI1 transcriptionally activates ALKBH5 expression, thereby promoting ALKBH5‐mediated m6A modification to downregulate NURR1 expression and ultimately stimulating the EMT‐like changes of RPE cells. We acknowledge that our serum‐based clinical data do not directly establish whether these molecular alterations occur in the RPE of AMD patients. However, independent studies have provided RPE‐specific evidence supporting the pathological relevance of each component of this axis: ALKBH5 is significantly upregulated in the RPE/choroid complex of clinical AMD specimens and in the RPE of laser‐induced CNV mice, and its overexpression in mouse RPE induces oxidative stress, autophagy dysregulation, and CNV progression [22]; NURR1 expression in primary RPE cells from human donor eyes declines with age, and NURR1 activation suppresses TNF‐α–induced RPE EMT [19]; for SPI1, although direct clinical data from AMD patient are unavailable, SPI1 is significantly upregulated in the RPE/choroid/sclera complex of aged and CNV mice, and its inhibition attenuates CNV progression [6], while SPI1 also critically regulates Aβ‐induced RPE oxidative stress [7]. These independent histological and in vivo findings corroborate the RPE‐specific pathological significance of the SPI1/ALKBH5/NURR1 axis.

Extensive studies have shown that small molecules and metabolites in the blood can serve as biomarkers for AMD and aid in its diagnosis and treatment [25, 26]. Notably, SPI1 expression is increased in the mouse model of diabetic macular edema [27]. Here, the expression of SPI1 in patients with AMD was significantly higher than that in the control group. ROC curve analysis showed that the AUC for SPI1 expression level in diagnosing AMD was 0.734 (95% CI: 0.652–0.806), with a cutoff value of 1.14 (sensitivity: 63.24%, specificity: 82.35%), suggesting that serum SPI1 has limited utility as an independent biomarker but offers complementary clinical information for AMD. Early diagnosis of AMD can be achieved by monitoring disease progression (regular OCT exams), while molecular biomarkers in body fluids can directly reflect early‐stage AMD‐associated inflammation, complement activation, or RPE injury, thereby providing molecular‐level evidence for asymptomatic early‐stage lesions [2]. Next, functional validation experiments showed that silencing SPI1 in the AMD cell model could reverse the EMT‐like changes, reduce ROS levels, and inhibit cell migration. A recent report has indicated that SPI1 upregulates the expression of EMT‐positively correlated genes ADH1B, MYH11, and PLN in endometriosis cells, thereby promoting cell invasion and migration [8]. In AMD mice, SPI1 overexpression promotes macrophage M1 polarization, induces the release of IL‐6/IL‐1β/TNF‐α, and facilitates choroidal neovascularization formation [26]. Notably, SPI1 is significantly upregulated in the RPE/choroid/sclera complex of aged and CNV mice, and SPI1 inhibition attenuates CNV progression [6]. Aβ1‐40 deposition induces SPI1 expression and nuclear translocation to activate the NOX4‐p22phox complex, thereby triggering oxidative stress and mitochondrial dysfunction in RPE cells [7]. Taken together, SPI1 knockdown prevents EMT‐like changes and inflammatory responses in RPE cells, thereby potentially inhibiting further RPE fibrosis and alleviating the progression of AMD.

Next, we demonstrated that SPI1 transcriptionally activated ALKBH5 expression, and SPI1 expression exhibited a positive correlation with ALKBH5. The m6A levels in the serum from AMD patients and in ARPE19 cells treated with TNF‐α were significantly decreased. Furthermore, in the AMD cell model, we found that after ALKBH5 overexpression, the EMT‐like changes in RPE cells were enhanced, and m6A level was decreased. Abnormal m6A levels are a key factor in retinal pathology [28, 29]. For instance, in the central nervous system, ALKBH5 knockout restores Lpin2 mRNA stability and enhances the survival rate of retinal ganglion cells following optic nerve injury [30]. ALKBH5 knockdown results in increased m6A levels and decreased A20 expression, which consequently leads to enhanced M1 polarization of retinal microglia in diabetic retinopathy [31]. ALKBH5 induces RPE dysfunction and choroidal neovascularization in AMD by demethylating PIK3C2B and activating the AKT/mTOR pathway [22]. Our study confirmed that ALKBH5‐mediated m6A demethylation downregulated the expression of NURR1 and thereby contributed to the pathogenesis of AMD, and that ALKBH5 expression exhibited a negative correlation with NURR1.

Finally, we performed functional validation by knocking down NURR1 in the AMD cell model and found that N‐cadherin expression was upregulated while E‐cadherin expression was downregulated, with increased ROS levels and cell migration, indicating the restoration of EMT‐like progression in RPE cells. NURR1 agonists can attenuate TNF‐α–induced EMT and migration, inhibit immune cell aggregation, reduce lipid accumulation, and improve retinal function [19]. In addition, NURR1 agonists can inhibit the NF‐κB/NLRP3 inflammasome axis, thereby reducing the loss of retinal ganglion cells [32]. Another report indicates that NURR1 can suppress EMT of hematopoietic stem cells and the release of proinflammatory cytokines, while also alleviating liver fibrosis [33]. In future studies, it is necessary to further investigate the mechanisms of NURR1 in RPE fibrosis and refine the mechanisms by which NURR1 exerts its effects in AMD.

Some limitations are as follows. The suboptimal AUC values indicate that SPI1 cannot be used as an independent diagnostic marker. Additional investigations are needed to confirm its clinical utility. TNF‐α–treated ARPE19 cells serve as an inflammation‐induced cell model with EMT‐like changes, which cannot fully recapitulate the complex pathological features of AMD. Further validation using AMD animal models or human ocular tissues is required in future studies. We only detected E‐cadherin and N‐cadherin but did not examine other canonical EMT markers, including vimentin, α‐SMA, fibronectin, and ZO‐1. In addition, we did not provide immunofluorescence or morphological evidence. Therefore, supplementary detection of the above markers is warranted in future studies to further strengthen our conclusions. Although the transcription factor SPI1 can regulate the expression of multiple downstream genes, only ALKBH5 was selected for investigation in this study. Similarly, as a demethylase, ALKBH5 can modify the expression of multiple genes via m6A modification, yet only NURR1 was investigated on in this study. The SPI1/ALKBH5/NURR1 axis may be involved in AMD pathogenesis. However, this study did not stratify AMD patients into dry and wet subtypes, and the relevant findings require further validation in subtype‐stratified cohorts in future investigations. The in vivo mechanism requires dedicated validation using RPE‐specific gene knockout and overexpression models, as well as tissue sections from AMD patients. Furthermore, other pathological mechanisms of AMD have not been explored. In future studies, we will further explore the downstream genes of SPI1 and ALKBH5, conduct animal experiments for validation, and provide a more solid theoretical basis for AMD treatment.

## 5. Conclusions

In conclusion, SPI1 is highly expressed in TNF‐α, and serum SPI1 has limited utility as an independent biomarker but offers complementary clinical information. SPI1 transcriptionally activates ALKBH5 expression, while ALKBH5 downregulates the expression of NURR1 by removing m6A modification and stimulates the EMT‐like changes of RPE cells.

**Nomenclature**
AMD: Age‐related macular degeneration
EMT: Epithelial–mesenchymal transition
RPE: Retinal pigment epithelial
SPI1: Spi‐1 proto‐oncogene
ALKBH5: AlkB homolog 5
NURR1: Nuclear receptor subfamily 4 group A member 2
RT‐qPCR: Reverse‐transcription quantitative polymerase chain reaction
ROC: Receiver operating characteristic
ROS: Reactive oxygen species
TNF‐α: Tumor necrosis factor‐α
DMEM: Dulbecco’s Modified Eagle Medium
FBS: Fetal bovine serum
GAPDH: Glyceraldehyde‐3‐phosphate dehydrogenase
RIPA: Radioimmunoprecipitation assay
BCA: Bicinchoninic acid
SDS‐PAGE: Sodium dodecyl sulfate polyacrylamide gel electrophoresis
PVDF: Polyvinylidene fluoride
PBS: Phosphate‐buffered saline
TBST: Tris‐buffered saline with Tween 20
IgG: Immunoglobulin G
ECL: Enhanced chemiluminescence
DCFH‐DA: 2′,7′‐Dichlorofluorescin diacetate
ChIP: Chromatin immunoprecipitation
m6A: N6‐methyladenosine
MeRIP‐qPCR: Methylated RNA immunoprecipitation‐quantitative polymerase chain reaction
ANOVA: Analysis of variance
AUC: Area under the curve
OCT: Optical coherence tomography

## Funding

No funding was received for this manuscript.

## Ethics Statement

This research was sanctioned by the Academic Ethics Committee of Dalian Medical University. All patients were fully informed of the purpose of the study and signed the informed consent form before sampling.

## Conflicts of Interest

The authors declare no conflicts of interest.

## Data Availability

Data will be made available on request.
